# Chemical Composition and Antimicrobial Activity of *Artemisia herba-alba* and *Origanum majorana* Essential Oils from Morocco

**DOI:** 10.3390/molecules24224021

**Published:** 2019-11-06

**Authors:** Ghita Amor, Lucia Caputo, Antonietta La Storia, Vincenzo De Feo, Gianluigi Mauriello, Taoufiq Fechtali

**Affiliations:** 1 Laboratory of Biosciences, Integrated and Molecular Functional Exploration, Faculty of Sciences and Techniques Mohammedia, 146 Mohammedia 20650, Morocco; amor.ghitaa@gmail.com; 2 Department of Agricultural Sciences, University of Naples Federico II, Via Università 100, 80055 Portici, Italy; alastoria@unina.it; 3 Department of Pharmacy, University of Salerno, Via Giovanni Paolo II 132, 84084 Fisciano, Italy; lcaputo@unisa.it

**Keywords:** essential oils, antimicrobial activity, chemical characterization, *Artemisia herba-alba*, *Origanum majorana*

## Abstract

Essential oils (EOs) are one of the most important groups of plant metabolites responsible for their biological activities. This study was carried out to study the chemical composition and the antimicrobial effects of *Artemisia herba-alba* and *Origanum majorana* essential oils against some Gram-positive and Gram-negative bacteria, and a fungal strain isolated from spoiled butter. The plants were collected in the region Azzemour of South West Morocco and the EOs, extracted by hydrodistillation, were analyzed by GC-MS. The antimicrobial activity was determined using the agar paper disc method. The main components of *A. herba-alba* EO were *cis*-thujone, *trans*-thujone and vanillyl alcohol; in *O. majorana* EO terpinen-4-ol, isopulegol and β-phellandrene predominated. Both essential oils exhibited growth inhibiting activities in a concentration-dependent manner on several microorganism species. Our results demonstrated that *O. majorana* and *A. herba-alba* EOs could be effective natural antibacterial agents in foods.

## 1. Introduction

Essential oils (EOs) are complex mixtures derived from various parts of plants with strong aromatic components such as terpenes. They are used in many fields such as medicine, cosmetic, and food industry [1,2]. The available literature reported that EOs possess, among others, significant antiseptic, antibacterial, antiviral, antioxidant, anti-parasitic, antifungal, and insecticidal activities [3].

At the moment, Morocco is considered as one of the principal suppliers and producers of some aromatic plants, such as *Artemisia herba-alba* Asso, *Mentha pulegium* L., *Lavandula stoechas* L., and *Rosmarinus officinalis* L. Moreover, these plants produce very high added value products contributing to the economic development of Morocco [4].

*Artemisia herba-alba*, *chih* in Arabic, belongs to the Asteraceae family; its essential oil is known for its antimicrobial, antioxidant, insecticidal, and antispasmodic activities. It is also used in traditional medicine as an antispasmodic and in treatment of diabetes mellitus [2,5].

*Origanum majorana* L. is a lamiaceous species, known for its antimicrobial, antioxidant, antidiabetic, and antitumoral activities [6]. In traditional medicine, the plant is used as an antiepileptic and a sedative drug [7].

The aim of the present study was to identify the components of *A. herba-alba* and *O. majorana* EOs from Morocco, and to evaluate their antimicrobial activity, against some Gram-positive and Gram-negative bacteria, and their antifungal efficacy.

## 2. Results

### 2.1. Essential Oil Yields and Composition

Hydrodistillation of the aerial parts of *A. herba alba* and of *O. majorana* resulted in pale yellow oils in 0.86% and 0.97% yield, on a dry mass basis, respectively. Table 1 and Table 2 report the percent composition of the essential oils; compounds are listed according to their elution on a HP-5MS column. Fifty-eight compounds were identified, 14 for *A. herba-alba*, and 44 for *O. majorana*, accounting for 97.6% and 97.8% of the total oil, respectively. In the essential oil from *A. herba-alba cis*-thujone (25.5%), *trans*-thujone (17.7%), vanillyl alcohol (11.5%), and *nor*-davanone (7.8%) are the main components. In the essential oil from *O. majorana,* terpinen-4-ol (34.1%), α-terpinene (19.2%), and terpineol (8.9%) are the main constituents.

### 2.2. Antimicrobial Activity

**Figure 1:**
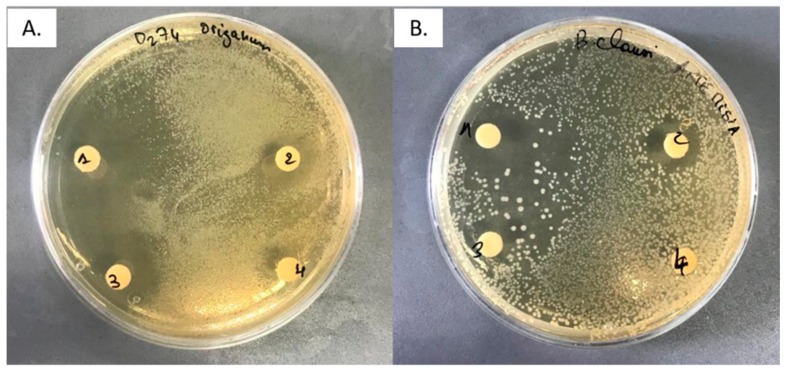
Representative antimicrobial activity of (**A**) *Origanum majorana* essential oil against *Brochothrix thermosphacta* D274 at the dose of 50, 40, 20, and 15 µL (from 1 to 4, respectively) and (**B**) *Artemisia herba-alba* essential oil against *Bacillus clausii* 2226 at the concentrations of 20, 15, 10, and 5 µL (from 1 to 4, respectively).

The antimicrobial activity of *A. herba-alba* and *O. majorana* essential oils was tested, at different concentrations, against 20 microorganisms, both Gram-positive and Gram-negative strains. Figure 1 shows a representative image of the antimicrobial activity. The EO of *A. herba-alba* showed inhibitory effects against 15 bacterial strains, the most sensitive being *Brochothrix thermosphacta* 7R1, *Bacillus clausii* 2226 and *Salmonella* Typhimurium; five strains resulted resistant to this EO: *Hafnia alvei* 53M, *Carnobacterium maltaromaticum* F1201, *Carnobacterium maltaromaticum* D1203, *Enterococcus faecalis* 226 and *Enterococcus faecalis* ES1 (Table 3). *O. majorana* essential oil showed a wider spectrum of activity as it was active against all microbial strains tested (Table 4).

Data analysis showed for the EO of *A. herba-alba* the same antimicrobial activity of tetracycline against *Streptococcus salivarius*, but higher than gentamicin, and exhibited stronger antimicrobial activity than both antibiotics against *Br. thermosphacta* D274, *B. clausii* 2226, and *S.* Typhimurium and lower antimicrobial activity than that of both antibiotics against *Staphylococcus* sp. GB1, *Staphylococcus saprophyticus* 3S, *Escherichia coli* 32, *Br. thermosphacta* 7R1, *Staphylococcus* sp. ES1, and *Serratia proteamaculans* 20P. On the other hand, the antimicrobial activity of *O. majorana* essential oil was stronger than both antibiotics against *Str. salivarius*, *E. coli* 32, *Br. thermosphacta* 7R1, *H. alvei* 53M, *Salmonella sp*. ES1, *Br. thermosphacta* D274, *B. clausii* 2226, *Ente. faecalis* 226, *S.* Typhimurium, and *Staphylococcus aureus.* The same antimicrobial activity as gentamicin was recorded against *Staph. saprophyticus* 3S, *C. maltaromaticum* H1201, *C. maltaromaticum* F1201, *Ent. faecalis* E21 and lower antimicrobial activity than both antibiotics against *Staph. sp* GB1 and *Listeria innocua* 1770.

### 2.3. Antifungal Activity

**Figure 2:**
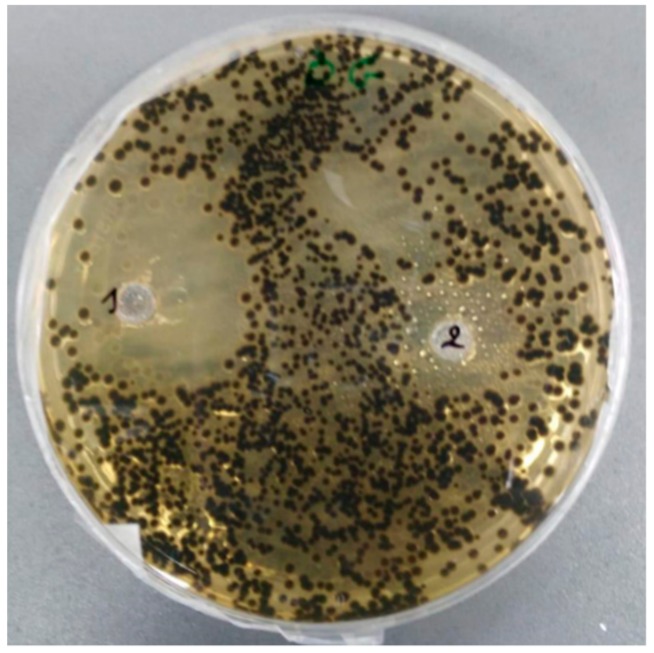
Antifungal activity of *A. herba-alba* (1) and *O. majorana* (2) essential oils against *Aspergillus niger* at the dose of 20 µL.

Table 5 reports the inhibition halos in (mm) at the dose of 20 µL of the two essential oils against *Aspergillus niger* isolated from the spoiled butter. This fungal strain was sensitive to both essential oils; the highest inhibitory activity was showed by *A. herba-alba* essential oil against *A. niger.* Figure 2 shows the antifungal activity of both EOs as determined in the same agar dish. However, diameters of inhibition halos were measured on two separated agar dishes.

## 3. Discussion

In our *A. herba-alba* essential oil oxygenated monoterpenes (57.3%) predominated, with *cis*-thujone (25.5%) and *trans*-thujone (17.7%) as the main constituents. Vanillyl alcohol (11.5%) and *nor*-davanone (7.8%) were in appreciable amounts.

These results agree with literature on the essential oil from *A. herba-alba* from different countries that evidenced *cis*- and/or *trans*-thujone as the principal constituents [8,9,10]. On the other hand, other studies showed eucalyptol (32.8%) as the main constituent of the *A. herba-alba* EO from Iran, and caryophyllene acetate (10.75%) for a Jordanian EO [11,12]. These compounds are totally absent in our essential oil. Camphor is reported as principal component in essential oil from Algeria and Tunisia (ranging between 19.6% and 50.5%) [13,14], but it is present in a low percentage in our sample (4.9%).

Moreover, other studies evidenced that davanone is one of the main constituents, with a percent greater than 10% [15,16]. In our EO, davanone and its derivative, *cis, threo*-davanafuran, accounted for 13.6% of the oil. Instead, this is the first report on the presence of vanillyl alcohol as one of the main constituents of this EO. Other studies reported camphor as the major component of the essential oil (17.8%–50.3%) [13,15,17,18,19] that, instead, is absent in our sample or chrysanthenone, present in our EO with its derivative, *iso*-chrysanthenyl acetate [20,21].

Monoterpenes predominated (91.1%) in the oil of *O. majorana*, both hydrocarbons and oxygenated compounds; sesquiterpenes accounted for 6.8%. The main components are terpinen-4-ol (34.1%), α-terpinene (19.2%), and terpineol (8.9%). Our results are in agreement with many studies that reported terpinen-4-ol among the principal constituents of the essential oil of *O. majorana* [22,23,24,25,26]. Moreover, α-terpinene was present in similar percentage (ranging from 11.08% to 12.72%) also in essential oils from Tunisia and Morocco [22,27]. Instead, in the EO from the Venuezelan Andes α-terpinene is reported in lesser percentages (3.6%) [26]. *trans*-Sabinene hydrate was reported as one of the principal components in other studies [28,29], in our sample its isomer was present in a low quantity (1.3%). Linalool, absent in our essential oil, is the main compound in the EO of *O. majorana* from Turkey with a percent of 88.01% [30]. Moreover, 4-terpinene and γ-terpinene were identified as the main components in *O. majorana* from Taiwan and Morocco, respectively [27,31].

Most microorganisms used in this study were sensitive to both essential oils, with the dose of 20 μL of EO sufficient to stop the growth of almost all tested Gram-positive and Gram-negative strains. In particular, *O. majorana* EO resulted more active, showing a wide spectrum of activity. On the other hand, the EO of *A. herba-alba* showed inhibitory effects against 15 bacterial strains.

The available literature reports the antimicrobial activity of *A. herba-alba* essential oil against *Staph. aureus*, *E. coli*, and *B. cereus* [23,32,33]. Moreover, several studies showed a great potential of *A. herba-alba* EO oil as an antibacterial agent against *Klebsiella pneumoniae*, *Listeria monocytogenes*, *Vibrio colerae*, and *S.* Typhimurium [34,35,36]. Our results showed variable antimicrobial and antifungal activity of the essential oil, being the inhibition zones in the range of 10–24 mm. Gram-positive bacteria resulted more sensitive to this EO. The Gram-positive *B. clausii* 2226 was the most sensitive tested strain, with the strongest inhibition zone (24.00 ± 1 mm). *B. clausii* was used as a model of spore-forming aerobic microorganism and our findings showed that our *A. herba-alba* EO is suitable to control the growth of this microorganism. It is well known that spore forming bacteria (also called thermoduric) are the main problem in pasteurized foods, both from the point of view of food spoilage and human intoxication. Gram-negative strains also displayed variable degree of susceptibility to this EO. The maximum activity was showed against the pathogen strain *S.* Typhimurium (17.7 ± 0.6), but *C. maltaromaticum* F1201, *C. maltaromaticum* D1203, *H. alvei* 53M, *Ent. faecalis* E21, and *Ent. faecalis* 226 resulted resistant, since no inhibition zone was observed. Due to the involvement of *S*. Typhimurium in the majority of food intoxication across the world, the antimicrobial capability of this EO could be of pivotal importance in the control of this microorganism in foods.

The antimicrobial activity of *O. majorana* essential oil appears to be similarly effective against both Gram-positive and Gram-negative microorganisms. These results agree with literature data [21,34,35]. Data of previous research showed that *O. majorana* essential oil was active against a large spectrum of different bacteria strains: *E. coli*, *Str. agalactiae*, *Shigella dysenteriae*, *Salmonella* Enteritidis, *Staph. aureus*, *Ent. faecalis*, *E. coli*, and *Klebsiella pneumoniae* [26,37].

In our study, all tested strains were sensitive to this essential oil, with the Gram-positive *S. aureus* the most sensitive with the greatest inhibition zone (32.2 ± 2.5 mm); the more sensitive Gram-negative was *S.* Typhimurium, with an inhibition zone of 29.7 ± 0.6 mm.

The antimicrobial activity of both essential oils could be related to their content in oxygenated monoterpenes, which constitute about 57.3% and 53.0% of the EOs of *A. herba alba* and *O. majorana*, respectively. Similar findings have been already previously reported [38,39].

The major components of *O. majorana* EO, e.g., terpinen-4-ol, α-terpinol and α-pinene, have been reported for their antimicrobial and antifungal properties [21]. Additionally, the main constituents of the EO of *A. herba-alba, cis*- and *trans*-thujone and vanillyl alcohol, have been reported for their antimicrobial, anti-inflammatory, and antioxidant activities [40,41]. Oxygenated monoterpenes exhibit high antimicrobial activity on whole cell and possess antifungal effects. These compounds diffuse into and damage cell membrane structures [42].

Our results showed high antifungal activity for both essential oils, with the highest inhibitory activity shown by the EO of *A. herba-alba* against *Aspergillus niger* (inhibition zone 23.6 ± 1.5 mm). These results are consistent with data previously reported [29,43].

## 4. Materials and Methods

### 4.1. Plant Material

The aerial parts of *A. herba-alba* and *O. majorana* were collected in the Azzemour region, South West Morocco, in June 2018, in flowering stage, and dried in the shade. The plants were identified by Prof. V. De Feo. A voucher specimen of each plant is stored in Department of Agricultural Sciences, University of Naples Federico II.

### 4.2. Essential Oil Extraction

One kilogram of *A. herba-alba* and *O. majorana* aerial parts was subjected to hydrodistillation for 3 h, according to the standard procedure described in the European Pharmacopoeia [44]. The oils were solubilized in *n*-hexane, filtered over anhydrous sodium sulphate and stored under N_2_ at +4 °C in the dark, until tested and analyzed.

### 4.3. GC-FID Analysis

Analytical gas chromatography was carried out on a Perkin-Elmer Sigma-115 gas-chromatograph (Perkin Elmer, Waltham, MA, USA) equipped with an FID and a data handling processor. The separation was achieved using a HP-5 MS fused-silica capillary column (30 m × 0.25 mm i.d., 0.25 µm film thickness). Column temperature: 40 °C, with 5 min initial hold, and then to 270 °C at 2 °C/min, 270 °C (20 min); injection mode splitless (1 µL of a 1:1000 *n*-hexane solution). Injector and detector temperatures were 250 °C and 290 °C, respectively. Analysis was also run by using a fused silica HP Innowax polyethylene glycol capillary column (50 m × 0.20 mm i.d., 0.25 µm film thickness). In both cases, helium was used as carrier gas (1.0 mL/min).

### 4.4. GC/MS Analysis

Analysis was performed on an Agilent 6850 Ser. II apparatus (Agilent, Roma, Italy), fitted with a fused silica DB-5 capillary column (30 m × 0.25 mm i.d., 0.33 µm film thickness), coupled to an Agilent Mass Selective Detector MSD 5973 (Agilent); ionization energy voltage 70 eV; electron multiplier voltage energy 2000 V. Mass spectra were scanned in the range 40–500 amu, scan time 5 scans/s. Gas chromatographic conditions were as reported in the previous paragraph; transfer line temperature, 295 °C.

### 4.5. Identification of the Essential Oil Components

Most constituents were identified by comparison of their Kovats retention indices (Ri) (determined relative to the tR of *n*-alkanes (C10–C35), with either those of the literature [45,46,47] and mass spectra on both columns or those of authentic compounds available in our laboratories by means of NIST 02 and Wiley 275 libraries [48]. The components relative concentrations were obtained by peak area normalization.

### 4.6. Antibacterial Assay

The antibacterial activity was evaluated in vitro, by means of the agar diffusion test on the plate. The activity of the essential oils was tested on the 20 microorganisms reported in Table 6. All of them belong to the collection of the Department of Agricultural Sciences, University of Naples Federico II.

Microbial strains were previously grown in TSB tryptone soya broth for 24 h. A volume of 0.1 mL of the microbial suspensions (about 1 × 10^8^ CFU/mL) was uniformly distributed on Nutrient agar plates in sterile conditions. Different amounts of essential oils were spotted on the inoculated plates: 50, 40, 20, 15, 10, and 5 µL for *O. majorana* and 20, 15, 10, and 5 µL for *A. herba-alba* essential oils. After 10 min, under sterile conditions, plates were then incubated at optimal growth condition culture of each strain. The antimicrobial activity was evidenced by measuring the diameter (in mm) of the zone of inhibition. Ethanol was used as the negative control; tetracycline (10 µg) and gentamycin (10 µg) were used as positive controls. Each experiment was carried out in three independent replicates and result is the average with standard deviation.

### 4.7. Antifungal Activity

The antifungal activity was evaluated in vitro, using the agar well diffusion method on the plates. The activity was tested against *Aspergillus niger* isolated from a spoiled butter sample and identified by phenotypic characteristics. The fungus was previously grown in TSA agar plates at 28 °C until spore formation. Then, 1 mL of a spore suspension in quarter strength Ringer solution, containing about 1 × 10^8^ spores per mL, was uniformly distributed on Nutrient agar plates in sterile conditions, then a hole was punched with sterile cork and 20 µL of each EO was introduced into the well; the plates were incubated at 28 °C for 4–5 days. Ethanol was used as the negative control. The antifungal activity was evaluated by measuring diameter of the inhibition area. Each experiment was carried out in three independent replicates and result is the average with standard deviation.

### 4.8. Statistical Analysis

Data of each experiment were statistically analyzed using GraphPad Prism 6.0 software (GraphPad Software Inc., San Diego, CA, USA), followed by comparison of means (one-way ANOVA) using Dunnett’s multiple comparisons test, at the significance level of *p* < 0.05.

## 5. Conclusions

The composition of *Artemisia herba-alba* and *Origanum majorana* essential oils growing in Morocco was analyzed and its antibacterial and antifungal activity investigated. The results indicated an important antimicrobial activity against different microorganisms especially from *O. majorana* essential oil. Thus, they can maybe be applied in food industry as natural preservatives, due to their antibacterial properties. Further organoleptic features and toxicological studies are required to prove the safety of the oils.

## Figures and Tables

**Table 1:** Chemical composition of *Artemisia herba-alba* essential oil.

Compound	%	Ki ^a^	Ki ^b^	Identification ^c^
*trans*-Arbusculone	4.5	1048		1,2
*cis*-Thujone	25.5	1079	1102	1,2,3
*trans*-Thujone	17.7	1111	1114	1,2,3
Camphor	4.9	1150	1146	1,2,3
*nor*-Davanone	7.8	1200	1231	1,2
*cis*-Chrysanthenylacetate	4.7	1231	1265	1,2
Undec-10-en-1-al	1.3	1261	1296	1,2
Cyclosativene	T	1342	1368	1,2
*cis, threo*-Davanafuran	5.8	1386	1415	1,2
Vanillyl Alcohol	11.5	1424	1447	1,2
*n*-Dodecanol	3.1	1445	1470	1,2
Isobornyl *n*-butyrate	4.9	1466	1491	1,2
<E>-Jasmolactone	3.4	1483	1491	1,2
Artedouglasia Oxide C	2.5	1496	1523	1,2
Total	97.6			
Oxygenated monoterpene	56.4			
Oxygenated sesquiterpenes	2.5			
Other compounds	38.7			

^a^ Kovats retention index on HP-5 MS column; ^b^ Kovats retention index on HP Innovax column; ^c^ Identification: 1 = Kovats retention index, 2 = mass spectrum, 3 = co-injection with pure compound; T = traces, less than 0.05%.

**Table 2:** Chemical composition of *Origanum majorana* essential oil.

Compound	%	KI ^a^	KI ^b^	Identification ^c^
α-Pinene	4.1	941	932	1,2,3
p-Cymene	2.6	950	1024	1,2,3
*iso*-Sylvestrene	0.6	952	1008	1,2,3
β-Pinene	0.2	975	974	1,2,3
α-Phellandrene	2.6	984	1002	1,2,3
δ-3-Carene	1.9	1008	1011	1,2
α-Terpinene	19.2	1021	1017	1,2,3
Limonene	0.1	1038	1029	1,2,3
1,8 Cineole	3.0	1047	1031	1,2,3
β-Ocimene	0.1	1061	1037	1,2,3
*cis*-Sabinene hydrate	1.3	1070	1070	1,2
Terpinen-4-ol	34.1	1096	1149	1,2,3
*endo*-Fenchyl-acetate	9.8	1114	1220	1,2
Pulegone	0.7	1122	1237	1,2
*trans*-Pinocarveol	0.3	1143	1139	1,2
Terpineol	8.9	1160	1133	1,2,3
*cis*-Limonene oxide	T	1188	1136	1,2
dihydro-Linalool	0.1	1191	1135	1,2
*cis*-Verbenol	T	1193	1141	1,2,3
Viridene	0.1	1199	1167	1,2
(E)-Isocitral	0.2	1205	1180	1,2
Thymol	0.2	1211	1290	1,2,3
Carvacrol	0.3	1220	1299	1,2,3
γ-Elemene	0.1	1233	1338	1,2,3
α-Terpinyl acetate	0.8	1242	1349	1,2
Eugenol	T	1271	1359	1,2,3
Neryl acetate	0.2	1274	1361	1,2
α-Copaene	T	1278	1376	1,2,3
Geranyl acetate	0.3	1293	1381	1,2,3
*iso*-Longifolene	0.1	1303	1390	1,2
(E)-Caryophillene	2.1	1314	1407	1,2,3
β-Duprezianene	T	1321	1422	1,2
β-Cedrene	T	1324	1420	1,2,3
β-Copaene	T	1327	1432	1,2,3
α-Guaiene	0.2	1332	1439	1,2,3
Aromadendrene	0.3	1336	1441	1,2,3
*allo*-Aromadendrene	1.3	1370	1460	1,2
Valencene	0.2	1401	1496	1,2,3
Caryophyllene oxide	T	1436	1583	1,2,3
Epiglobulol	T	1445	1590	1,2
(-)-Spathulenol	T	1453	1578	1,2
β-Atlanthol	1.6	1464	1608	1,2,3
Rosifoliol	0.1	1485	1600	1,2
Cubenol	T	1497	1646	1,2
Total	97.8			
Monoterpene hydrocarbons	33.1			
Oxygenated monoterpene	57.9			
Sesquiterpene hydrocarbons	5.1			
Oxygenated sesquiterpenes	1.7			

^a^ Kovats retention index on HP-5 MS column; ^b^ Kovats retention index on HP Innovax column; ^c^ Identification: 1 = Kovats retention index, 2 = mass spectrum, 3 = co-injection with pure compound. T = traces, less than 0.05%.

**Table 3:** Antibacterial activity of the essential oil of *A. herba-alba*.

Strain	Control		Essential Oil			
	Gentamicin	Tetracyclin	5 μL	10 μL	15 μL	20 μL
*B. clausii 2226*	11.0 ± 1.0	16.3 ± 1.5	10.3 ± 0.6	14.7 ± 0.6	19.7 ± 1.5 ^a,D^	24.0 ± 1.0 ^a,A^
*Br. thermosphacta* 7R1	18.3 ± 1.5	19.3 ± 1.2	na	6.0 ± 0.0	6.0 ± 0.1	12.3 ± 0.6
*Br. thermosphacta* D274	6.0 ± 0.0	8.7 ± 1.2	6.0 ± 0.0	11.7 ± 0.6 ^a,C^	14.7 ± 0.6 ^a,A^	17.7 ± 0.6 ^a,A^
*C. maltaromaticum* 9P	6.0 ± 0.0	24.3 ± 1.2	6.0 ± 0.0	9.0 ± 1.0 ^b^	11.7 ± 0.6 ^a^	12.3 ± 0.6 ^a^
*C. maltaromaticum* D1203	6.0 ± 0.0	22.3 ± 0.6	na	na	na	na
*C. maltaromaticum* F1201	6.0 ± 0.0	23.3 ± 1.5	na	na	na	na
*C. maltaromaticum* H1201	10.0 ± 0.0	na	na	na	na	6.0 ± 0.0 ^A^
*E. coli* 32	14.7 ± 0.6	18.7 ± 1.2	na	na	6.0 ± 0.0	6.0 ± 0.0
*Ent. faecalis* 226	6.0 ± 0.0	9.0 ± 1.0	na	na	na	na
*Ent. faecalis* E21	6.0 ± 0.0	14.7 ± 0.6	na	na	na	na
*H. alvei* 53M	11.7 ± 1.5	9.7 ± 0.6	na	na	na	na
*L. innocua* 1770	25.3 ± 0.6	20.3 ± 1.5	na	na	na	6.0 ± 0.0
*P. fragi* 6P2	14.7 ± 0.6	17.0 ± 1.0	na	6.0 ± 0.0	9.3 ± 0.6	13.3 ± 2.1
*Staph. aureus*	6.0 ± 0.0	15.3 ± 0.6	na	na	na	6.0 ± 0.0
*S.* Typhimurium	9.7 ± 0.6	12.7 ± 1.2	na	6.0 ± 0.0	14.0 ± 1.7 ^b^	17.7 ± 0.6 ^a,B^
*Serr. proteamaculans* 20P	12.3 ± 0.6	24.3 ± 1.2	na	6.0 ± 0.0	7.7 ± 0.6	10.3 ± 0.6
*Str. salivarius*	6.0 ± 0.0	18.7 ± 1.2	na	6.0 ± 0.0	14.0 ± 1.0 ^a^	18.3 ± 1.5 ^a^
*Staph. saprophyticus* 3S	24.0 ± 1.0	29.0 ± 3.6	na	na	6.0 ± 0.0	6.0 ± 0.0
*Staph. sp.*ES1	19.3 ± 1.2	29.3 ± 1.2	na	6.0 ± 0.0	8.3 ± 0.6	10.3 ± 0.6
*Staph.sp.*GB1	21.3 ± 1.2	27.7 ± 2.5	6 ± 0.0	11.3 ± 1.2	14.7 ± 0.6	17 ± 1.0

Data represent the diameter inhibition (in mm). Results are the mean of three repetitions ± standard deviation (SD) of the inhibition zone. na = not active. Dunnett’s test vs. Gentamicin (^a,b,c,d^) or Tetracycline (^A,B,C,D^): ^a,A^ *p* < 0.0001; ^b,B^ *p* < 0.001; ^c,C^ *p* < 0.01; ^d,D^ *p* < 0.05. *B.: Bacillus*; *Br.: Brochothrix*; *C.: Carnobacterium*; *E.: Enterococcus*; *Staph.: Staphylococcus*; *L.: Listeria*; *E.: Escherichia*; *H.: Hafnia*; *P.: Pseudomonas*; *S.: Salmonella; Serr.: Serratia*; *Str.: Streptococcus*.

**Table 4:** Activity of the essential oil of *O. majorana*.

Strain	Control		Essential Oil					
	Gentamicin	Tetracyclin	5 µL	10 µL	15 µL	20 µL	40 µL	50 µL
*B. clausii* 2226	11.0 ± 1.0	16.3 ± 1.5	na	6.0 ± 0.0	15.3 ± 0.6^b^	23.3 ± 1.5 ^a,B^	24.7 ± 0.6 ^a,B^	28.3 ± 1.5 ^a,B^
*Br. thermosphacta* D274	6.0 ± 0.0	8.7 ± 1.2	10.6 ± 0.6^d^	13.3 ± 1.2 ^a^	18.3 ± 0.6 ^a,B^	23.0 ± 1.7 ^a,B^	24.3 ± 1.2 ^a,B^	26.3 ± 1.2 ^a,B^
*Br. thermosphacta* 7R1	18.3 ± 1.5	19.3 ± 1.2	11.3 ± 1.2	15.3 ± 0.6	18.0 ± 0.0	20.3 ± 0.6^*^	20.0 ± 0.0	21.3 ± 0.6^c,D^
*C. maltaromaticum* 9P	6.0 ± 0.0	24.3 ± 1.2	na	na	na	6.0 ± 0.0	6.0 ± 0.0	9.3 ± 0.6^a^
*C.maltaromaticum* H_1_201	10.0 ± 0.0	na	6.0 ± 0.0^A^	6.0 ± 0.0 ^A^	8.7 ± 1.2 ^A^	9.7 ± 0.6 ^A^	9.3 ± 0.6 ^A^	9.7 ± 0.6 ^A^
*C. maltaromaticum* D_1_203	6.0 ± 0.0	22.3 ± 0.6	na	na	na	6.0 ± 0.0	9.3 ± 0.6 ^a^	13.3 ± 1.5 ^a^
*C. maltaromaticum* F_1_201	6.0 ± 0.0	23.3 ± 1.5	na	na	na	6.0 ± 0.0	6.0 ± 0.0	6.0 ± 0.0
*E. coli* 32	14.7 ± 0.6	18.7 ± 1.2	9.7 ± 0.6	10.7 ± 1.2	17.7 ± 0.6	20.0 ± 0.0 ^a^	24.3 ± 1.2 ^a,B^	26.7 ± 0.6 ^a,B^
*Ent. faecalis* 226	6.0 ± 0.0	9.0 ± 1.0	na	na	na	6.0 ± 0.0	6.0 ± 0.1	9.7 ± 0.7
*Ent. faecalis* E21	6.0 ± 0.0	14.7 ± 0.6	na	na	na	6.0 ± 0.0	6.0 ± 0.0	6.0 ± 0.0
*H. alvei* 53M	11.7 ± 1.5	9.7 ± 0.6	8.3 ± 0.6	10.3 ± 0.6	11.7 ± 0.6 ^C^	12.7 ± 0.6 ^A^	15.0 ± 0.0 ^b,A^	20.3 ± 0.6 ^a,B^
*L. innocua* 1770	25.3 ± 0.6	20.3 ± 1.5	na	6.0 ± 0.0	9.3 ± 0.6	11.7 ± 0.6	12.3 ± 0.6	13.7 ± 1.5
*P. fragi* 6P2	14.7 ± 0.6	17.0 ± 1.0	na	na	6.0 ± 0.0	6.0 ± 0.0	6.0 ± 0.0	9.3 ± 0.6
*Staph. aureus*	6.0 ± 0.0	15.3 ± 0.6	10.7 ± 0.6 ^d^	11.7 ± 1.5 ^c^	16.3 ± 1.5 ^a^	24.3 ± 2.1 ^a,B^	27.7 ± 0.6 ^a,B^	32.3 ± 2.5 ^a,B^
*S.* Typhimurium	9.7 ± 0.6	12.7 ± 1.2	7.7 ± 2.1	11.7 ± 0.6	14.0 ± 1.7 ^d^	17.3 ± 1.2 ^c,D^	23.3 ± 2.9 ^a,B^	29.7 ± 0.6 ^a,B^
*Serr.proteamaculans* 20P	12.3 ± 0.6	24.3 ± 1.2	na	na	na	6.0 ± 0.0	16.3 ± 1.5^a^	19.3 ± 1.2^a^
*Str. salivarius*	6.0 ± 0.0	18.7 ± 1.2	9.7 ± 0.6 ^c^	11.3 ± 0.6 ^a^	13.0 ± 1.0 ^a^	19.3 ± 1.2 ^a^	20.7 ± 1.2 ^a^	24.3 ± 1.2 ^a,B^
*Staph. saprophyticus* 3S	24.0 ± 1.0	29.0 ± 3.6	9.9 ± 1.0	11.7 ± 0.6	19.3 ± 1.2	20.3 ± 0.6	21.0 ± 1.0	24.3 ± 1.2
*Staph.sp.* ES1	19.3 ± 1.2	29.3 ± 1.2	6.0 ± 0.0	6.0 ± 0.0	14.7 ± 7.6	18.7 ± 1.2	20.0 ± 0.0	21.0 ± 1.0
*Staph.sp.*GB1	21.3 ± 1.2	27.7 ± 2.5	na	na	5.7 ± 0.6	10 ± 0.0	14.7 ± 0.6	19.3 ± 1.2

Data represent the diameter inhibition(in mm). Results are the mean of three repetitions ± standard deviation (SD) of the inhibition zone. na = not active. Dunnett’s test vs. Gentamicin (^a,b,c,d^) or Tetracycline (^A,B,C,D^): ^a,A^ *p* < 0.0001; ^b,B^ *p* < 0.001; ^c,C^ *p* < 0.01; ^d,D^ *p* < 0.05. *B.: Bacillus*; *Br.: Brochothrix*; *C.: Carnobacterium*; *E.: Enterococcus*; *Staph.: Staphylococcus*; *L.: Listeria*; *E.: Escherichia*; *H.: Hafnia*; *P.: Pseudomonas*; *S.: Salmonella; Serr.: Serratia*; *Str.: Streptococcus*.

**Table 5:** Antifungal activity of *A. herba-alba* and *O. majorana* essential oils.

	*Aspergillus niger*
*Artemisia herba-alba*	23.6 ± 1.5
*Origanum majorana*	14.0 ± 1.0

Data represent the diameter inhibition (in mm). Results are the mean of three repetitions ± standard deviation (SD) of the inhibition zone.

**Table 6:** Source and optimal growth conditions of microorganisms.

Gram	Microorganism	Source	Growth Conditions
**Positive**	*B. clausii* 2226	Supplement	TSB 24h at 30 °C
	*Br. thermosphacta* 7R1	Meat	TSB 24h at 20 °C
	*Br. thermosphacta* D274	Meat	TSB 24h at 20 °C
	*C. maltaromaticum* 9P	Meat	TSB 24h at 20 °C
	*C. maltaromaticum* D1203	Meat	TSB 24h at 25 °C
	*C. maltaromaticum* F1201	Meat	TSB 24h at 25 °C
	*C. maltaromaticum* H1201	Meat	TSB 24h at 25 °C
	*Ent. faecalis* 226	Milk	TSB 24h at 30 °C
	*Ent. faecalis* E21	Milk	TSB 24h at 30 °C
	*Staph. aureus*	Meat	TSB 24h at 37 °C
	*Staph. saprophyticus* 3S	Fermented meat	TSB 24h at 37 °C
	*Staph.* sp. ES1	Fermented meat	TSB 24h at 37 °C
	*Staph.* sp. GB1	Fermented meat	TSB 24h at 37 °C
	*L. innocua* 1770	Milk	TSB 24h at 30 °C
	*E. coli* 32	Meat	TSB 24h at 37 °C
	*Str. salivarius*	Milk	TSB 24h at 30 °C
**Negative**	*H.alvei* 53M	Meat	TSB 24h at 30 °C
	*Pseud. fragi* 6P2	Meat	TSB 24h at 20 °C
	*S.* Typhimurium	Chicken meat	TSB 24h at 30 °C
	*Serr.proteamaculans*20P	Meat	TSB 24h at 25 °C

*B.: Bacillus; Br.: Brochothrix; C.: Carnobacterium; E.: Enterococcus; Staph.: Staphylococcus; L.: Listeria; E.: Escherichia; H.: Hafnia; P.: Pseudomonas; S.: Salmonella; Serr.: Serratia; Str.: Streptococcus.*
